# Imaging of Benign Tumors of the Osseous Spine

**DOI:** 10.5334/jbsr.1380

**Published:** 2018-01-31

**Authors:** Hend Riahi, Meriem Mechri, Maher Barsaoui, Mouna Bouaziz, Filip Vanhoenacker, Mohamed Ladeb

**Affiliations:** 1 Institut Kassab d’orthopédie, TN; 2 la rabta, TN; 3 AZ Sint-Maarten and University (Hospital) Antwerp/Ghent, BE

**Keywords:** Spine, Bone tumors, Radiograph, CT, MRI

## Abstract

The purpose of this paper is to present an overview of the imaging features of the most prevalent benign bone tumors involving the spine. Benign tumors of the osseous spine account approximately for 1% of all primary skeletal tumors. Many lesions exhibit characteristic radiologic features. In addition to age and location of the lesion, radiographs are an essential step in the initial detection and characterization but are limited to complex anatomy and superposition. CT and MR imaging are often mandatory for further characterization, assessment of local extension and guiding biopsy.

## Introduction

As most of benign tumors of the osseous spine are asymptomatic, their real incidence is unknown. The prevalence is estimated for less than 10% of all spinal tumors [1]. Differentiation of benign tumors from malignant tumors is of paramount importance, because of the difference in management. Imaging may play an important role in making a correct diagnosis. Radiographs can help to detect and characterize almost 80% of the benign tumors. In addition, Computed Tomography (CT) and Magnetic Resonance Imaging (MRI) are extremely useful tools for further characterization and surgical planning. The aim of this paper is to review the imaging characteristics of the most common benign primary spinal tumors.

## Osteoid osteoma

**Figure 1:**
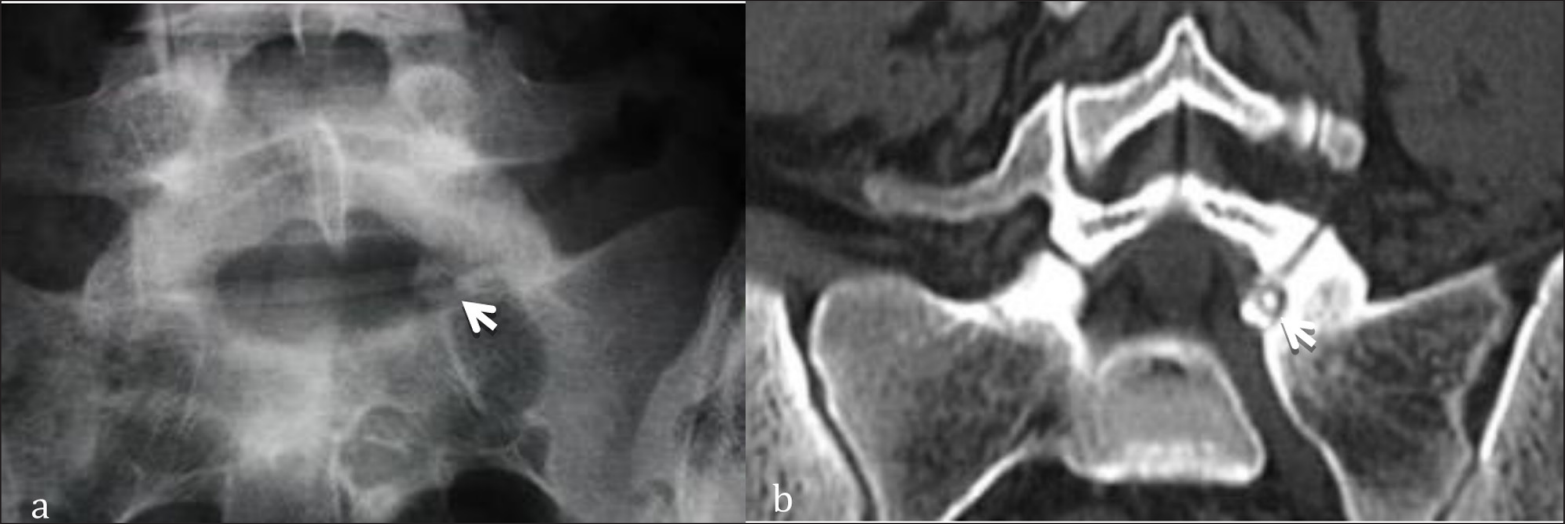
Osteoid osteoma of L5. Radiograph **(a)** shows focal lucency in the neural arch. CT scan **(b)** shows the calcified nidus (arrow).

Osteoid osteoma (OO) is characterized by the formation of a nidus of vascularized osteoid tissue surrounded by a halo of reactive sclerotic bone. The average size of the nidus is less than 1.5 cm. It commonly occurs in the second decade. OO accounts for 1% of all spinal tumors. The lumbar spine is most commonly affected (59%), followed by the cervical, thoracic, and sacral spine [2]. Most spinal OO are located in the posterior elements of the vertebra. Stiff and painful scoliosis is the most typical presentation (20–70% of cases) [3]. Muscular spasm may result in scoliosis, which is initially non-rotational. The lesion usually lies at the apex of the concavity. Spinal OO should be suspected in any case of atypical scoliosis (left convex thoracic scoliosis or right convex lumbar scoliosis). Hyperlordosis, kyphoscoliosis and torticolis may also occur [4]. Other clinical features consist of nerve root irritation, night pain and dramatic pain relief with aspirin or non-steroidal anti-inflammatory drugs (14%–90%) [5]. Macroscopically, OO appears as a reddish, round-shaped lesion. Microscopicably, the nidus is composed of well-organized, interconnected trabecular bone with a background of vascularized fibrous connective tissue. Radiographs show a central lucency, with variable degree of mineralization and surrounding sclerosis [2]. Due to superposition of surrounding sclerosis, the nidus may be difficult to detect radiographically (Figure 1a). Additional radiographic signs include regional osteoporosis, solid or laminated periosteal reaction and scoliosis. Bone scintigraphy may be useful for detection of a radiographically occult nidus [7]. OO typically has early avid uptake on triple-phase scintigraphy [7]. CT is the modality of choice in the diagnosis of spinal OO. CT typically shows a small rounded low-attenuation nidus, surrounded by extensive sclerosis. The nidus may contain a central, irregular or ring-shaped nucleus of mineralization (Figure 1b). The MRI appearance varies along with the amount of calcification within the nidus, size of the fibrovascular zone, amount of reactive sclerosis and bone marrow edema [8]. On non-enhanced MRI, the nidus is best visualized on T2-WI sequences where it appears as a hypointense lesion surrounded by bone marrow edema. The nidus is of intermediate signal intensity on T1-WI. Focal signal void due to matrix mineralization is occasionally seen. Dynamic gadolinium-enhanced MRI improves detection of the nidus [9]. MRI is particularly useful to evaluate the effect of the OO on the spinal canal and cord [10]. The main differential diagnoses are osteoblastoma, osteoblastic metastasis, infection and reactive sclerosis caused by facet degeneration [6].

Although spontaneous resolution has been reported rarely, prompt treatment is preferred. Indeed, treatment delay in the growing child may result in a permanent structural scoliosis [11]. Alternative treatment options to excision include:

◽ Percutaneous curettage of the nidus under CT guidance.
◽ Percutaneous CT-guided thermo-coagulation. This technique achieves the same clinical outcome as the operative excision with significantly lower costs.
◽ Percutaneous CT-guided interstitial laser thermotherapy [12].

Regrowth of OO is generally due to incomplete removal rather than multiple nidus. CT is the preferred technique for follow-up after treatment.

## Osteoblastoma

**Figure 2:**
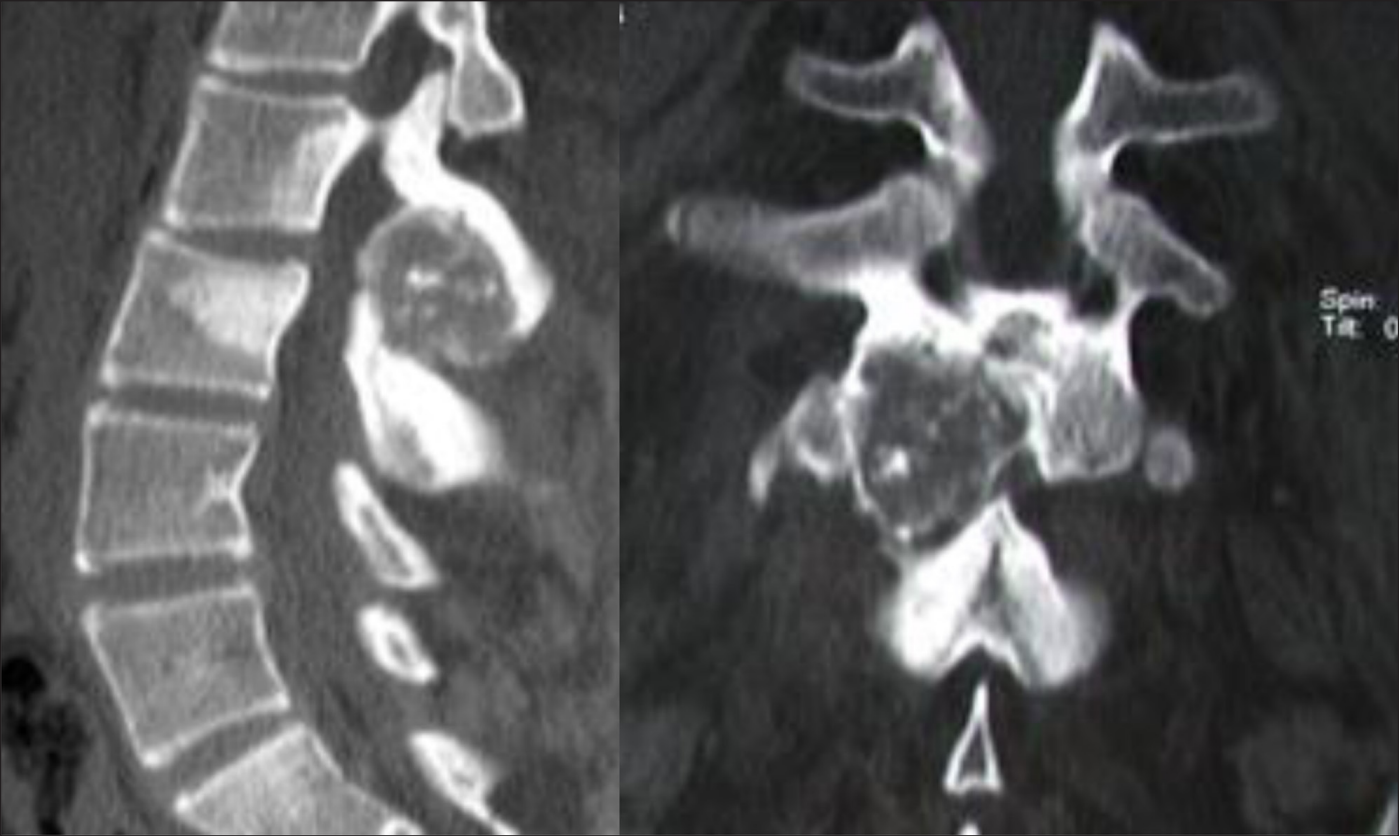
Osteoblastoma of L2. CT scan shows an expansive osteolytic lesion of the L2 neural arch with central calcification, sclerosis of the body and the neural arch of L2 and L3.

**Figure 3:**
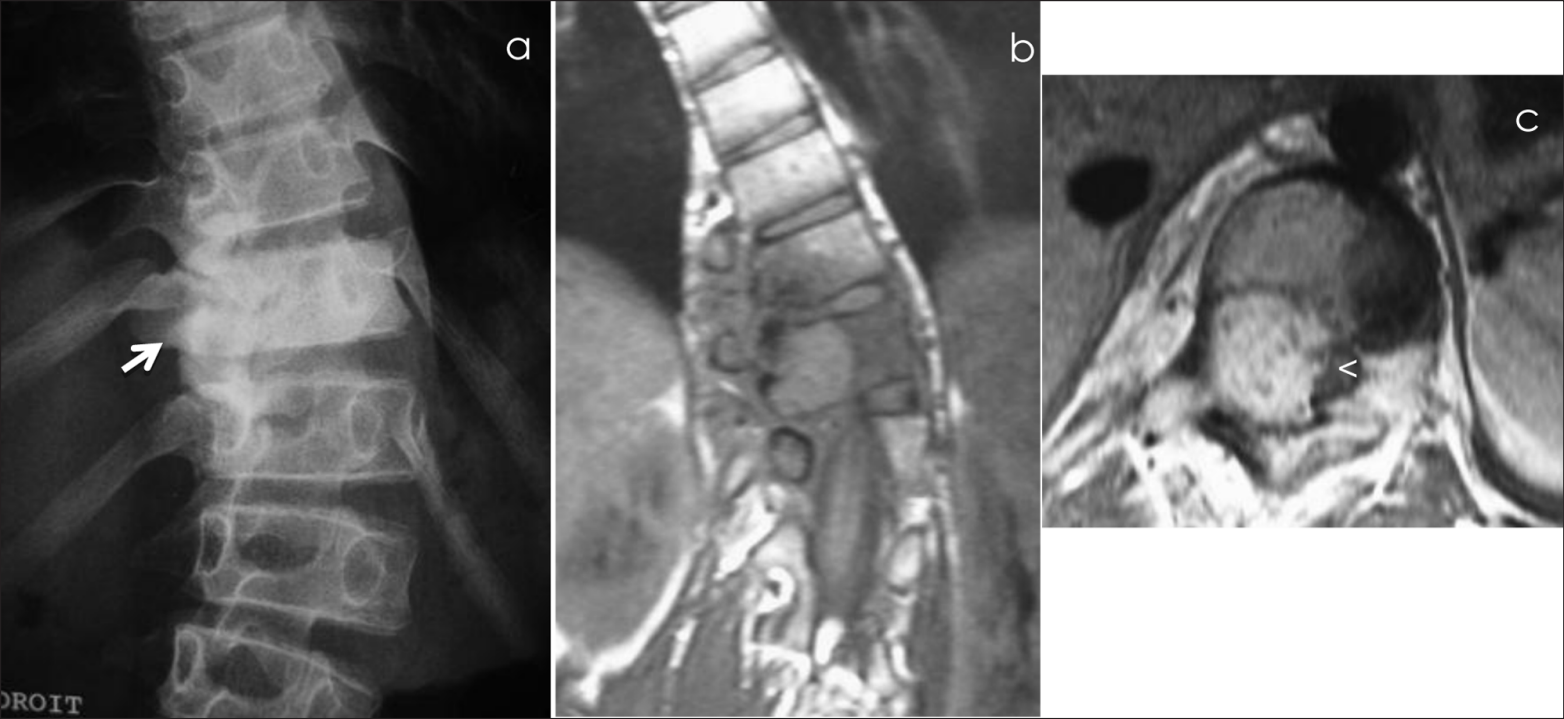
Osteoblastoma of T11. Radiograph **(a)** shows scoliosis and focal sclerosis of T11 (arrow). Coronal T1-WI **(b)** and axial T1-WI after gadolinium contrast administration **(c)** shows a lesion of the neural arch of T11, intralesional calcification and adjacent bone marrow edema of the vertebral bodies of T10 and T11. Note the compression of spinal cord (arrowhead).

Osteoblastoma is a benign bone forming tumour which is histologically similar to OO. In comparison to osteoid osteoma, osteoblastomas tend to be larger in size (>2 cm), often present with cortical breakthrough and a soft tissue mass, and may rarely undergo malignant transformation [13,14]. Spinal osteoblastomas account for 30–40% of all osteoblastomas. They most commonly occur in the second to third decade, which is slightly later than the usual age for OO. It is more common in males than females (ratio of 2–2.5/1). Osteoblastoma originates in the neural arch and then extends to the vertebral body. Thoracic, cervical, and lumbar segments are equally affected. The most common symptom is pain. Unlike OO, pain associated with an osteoblastoma is more generalized, and less likely to be relieved by salicylates. Scoliosis has also been described in osteoblastoma. Neurological symptoms are estimated to occur in more than half of all patients with osteoblastoma due to either pathological fractures or soft tissue extension [15]. Radiological presentation of osteoblastoma may vary. Osteoblastoma may be similar to osteoid osteomas, with a radiolucent nidus and surrounding sclerotic changes (Figure 2). The aggressive variant radiographs may show bone expansion with matrix calcifications, cortical bone destruction, paravertebral and epidural extension [16]. CT is useful for precise assessment of the location of the tumor and the extra-osseous involvement. MRI is the most useful technique for evaluation of the potential mass effect of the tumor on neural elements. The imaging characteristics on MRI are generally nonspecific and depend on the degree of tumor mineralization. On T1-WI, the lesion is hypointense with mixed signal on T2-WI and an intense enhancement (Figure 3).

Surrounding bone marrow edema in adjacent vertebrae, paraspinal soft tissues and ribs enhances after contrast administration, often resulting in overestimation of size of lesion. Adjacent bone remodeling at the level of the articular facet may present as facet hypertrophy [16]. Technetium-99 bone scintigraphy reveals avid uptake [17].

Resection is the best treatment option for osteoblastoma, but local recurrence, especially after incomplete resection, has been reported in approximately 50%. Some studies have found beneficial effects of radiotherapy following subtotal excision [18].

## Osteochondroma

Osteochondroma (OC) is the most common benign tumor of the bone. Osteochondroma of the spine is rare and comprises only 1.3–4.1% of all osteochondromas [19]. Solitary lesions are more common at the cervical spine (50–58%) whereas multiple lesions typically occur at the thoracolumbar region. Spinal osteochondromas arise from excessive cartilaginous tissue of secondary ossification centers in the posterior elements but may also originate from the vertebral body [20]. Benign osteochondromas do not grow after skeletal maturity, and they usually manifest clinically during the second to third decades of life [21]. They are more common in males than females by three to one.

**Figure 4:**
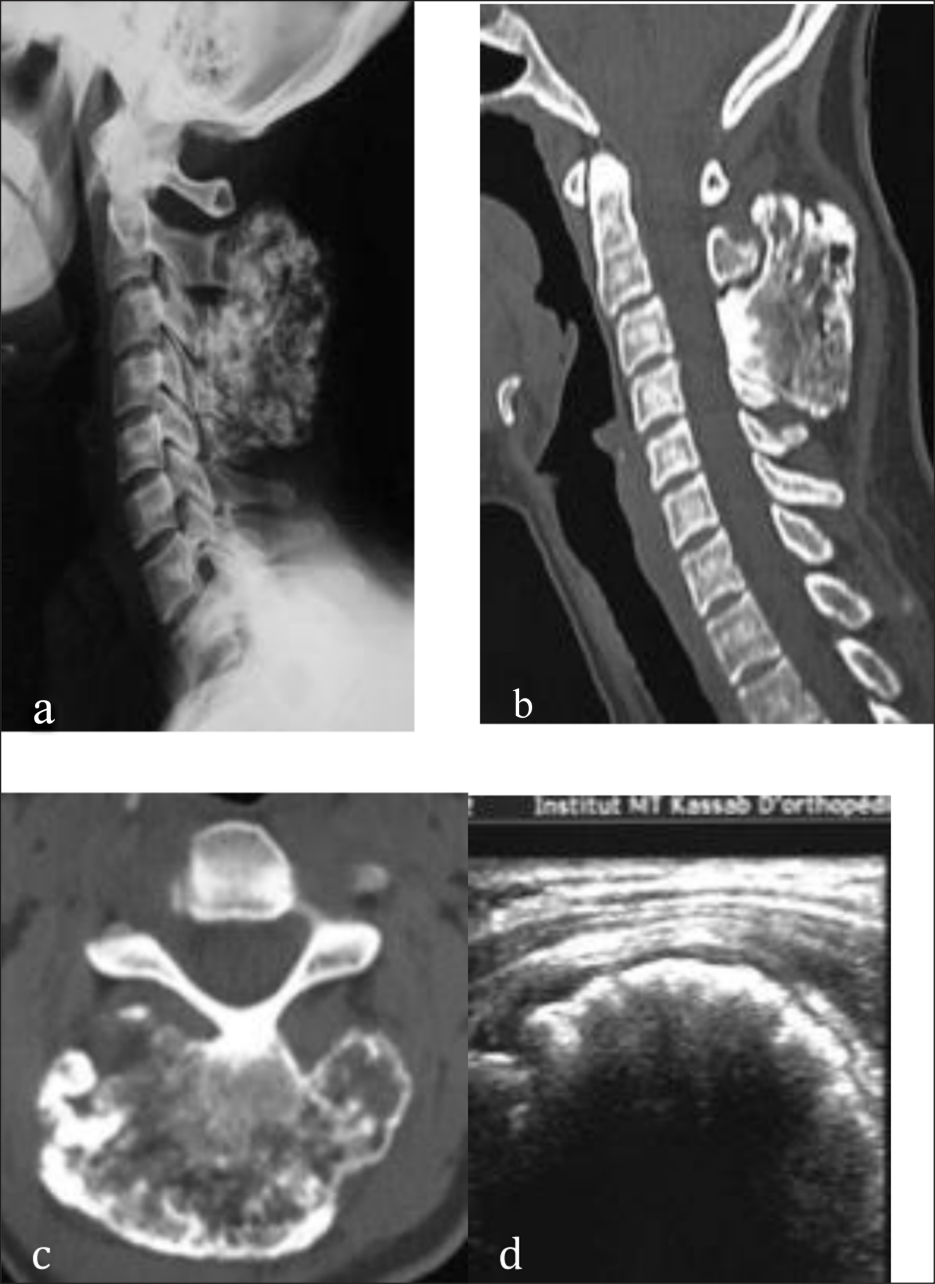
Osteochondroma of the cervical spine. Radiograph **(a)** shows an osseous mass located in the spinous processes of C3 and C4. CT scan **(b, c)** shows continuity of the osteochondroma with the spinous processes of C3 and C4. US **(d)** demonstrates a thin cartilaginous cap.

Spinal osteochondromas may be part of Hereditary Multiple Exostosis Syndrome. The clinical manifestations of osteochondroma vary widely, ranging from back pain to neurological deficits mimicking myelopathy or radiculopathy [21,22]. Because of the complex anatomy of the spine, it is difficult to diagnose spinal osteochondromas by radiographs. (Figure 4a). Therefore, spinal osteochondromas are better evaluated by CT and MRI. CT enables precise localization of the lesion (Figure 4b, c) and its relationship to the central canal and neuroforamina of the spine. On MRI, the signal of osteochondromas varies depending on the size of the lesion, the amount of bone marrow and degree of cartilage calcification. The medullary and cortical components of the osteochondroma mimic normal bone. The central fatty marrow appears hyperintense on T1-WI and T2-WI with a peripheral hypointense rim representing the cortex. The signal of the cartilage cap differs depending on degree of mineralization and should not exceed 2 cm. Peripheral and septal contrast enhancement may be seen after intravenous gadolinium contrast administration [2]. MRI evaluates better the relationship with the spinal cord and nerve roots. Malignant transformation should be suspected in case of recurrence after total resection, growth after maturation of the skeleton system, increase of the lesion’s size, altered surface delineation, intralesional osteolysis, destruction of adjacent bones and soft-tissue masses containing scattered or irregular calcifications.

When the tumor causes pain or neurological complications due to compression, or if the diagnosis is indeterminate, osteochondroma should be excised at its base [21].

## Hemangiomas

Vertebral hemangiomas are the most common benign tumors in the spine, accounting for 2% of skeletal benign tumors. They are considered as dysembryogenetic disturbances, affecting the proper differentiation of blood vessels [23]. There is no sex predominance. It can occur at any age, but mostly after the fourth or fifth decade of life. Vertebral hemangiomas are most frequently in the thoracic spine, followed by the lumbar region, and rarely in the cervical and sacral segments [24].

Most vertebral hemangiomas are asymptomatic. The patients present with pain, spinal cord or nerve root compression in case of aggressive hemangiomas.

Histopathologically, hemangiomas are predominantly composed of vascular lined spaces and nonvascular components that may include adipose tissue, smooth muscle, fibrous tissue, bone, hemosiderin and thrombus [25].

**Figure 5:**
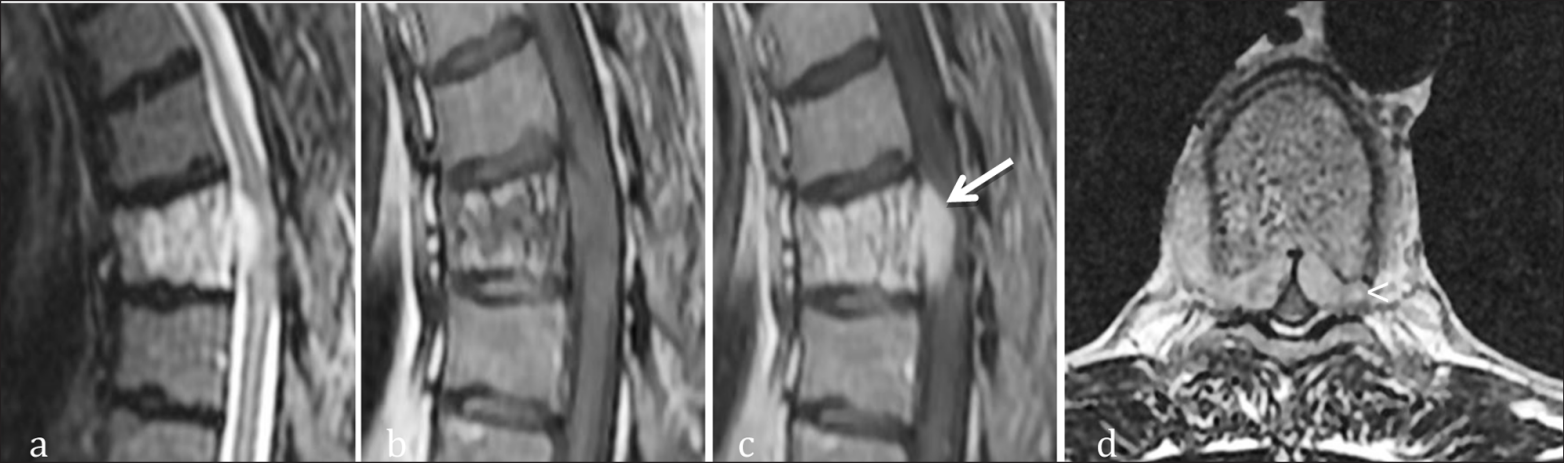
Aggressive hemangioma of T5. Sagittal T2-WI **(a)**, Sagittal T1 WI **(b)** and sagittal **(c)** and axial **(d)** T1-WI after gadolinium contrast administration **(c)** MR images shows an aggressive hemangioma of T6 with extension into the anterior epidural space (arrow) and soft tissues (arrowhead).

On radiographs, hemangiomas cause rarefaction of the trabeculae which may be thickened causing vertical striation. On CT, these vertical striations interspersed with fatty attenuation cause the so-called *polka dot* on axial images or *corduroy* appearance on coronal or sagittal reformatted images respectively. On MRI, fatty hemangiomas represent inactive forms. Low T1 signal intensity indicates a more active lesion with the potential to compress the spinal cord. Haemangiomas show avid enhancement. Aggressive hemangiomas involve the entire vertebral body with extension to the neural arch, cortical expansion, irregular honeycombing, soft tissue mass and contrast enhancement (Figure 5). These aggressive variants may be differentiated from metastasis by using chemical-shift imaging because they lose signal on out-of-phase images. Another differential diagnosis for an aggressive hemangioma is bone lymphoma. CT of lymphoma usually reveals cortical permeation with extension into surrounding soft tissues [26].

In symptomatic spinal hemangiomas local control and long-term survival can be obtained after resection [27].

## Giant cell tumors

Spinal giant cell tumors (GCT) are quite rare and comprise less than 5% of the primary bone tumors of the spine [28]. GCTs are more frequently found in women (female/male ratio 2.5/1) and affect patients in their second to fourth decades of life [29]. Being an aggressive bone tumor, local or distant spread predominantly to the lungs may occur and the recurrence rate following surgical excision is high. Most lesions occur in the sacrum (1.3%–9.3%), followed in order of decreasing frequency by the thoracic, cervical, and lumbar segments [30]. GCTs typically involve the vertebral body and may extend into the posterior elements and paraspinal tissues. Adjacent disks and vertebrae can be involved [31].

Patients typically present with pain, neurologic deficits (72%) such as radicular pain and motor weakness from nerve root or spinal cord compression [31].

Histopathologically, GCTs consists of abundant osteoclastic giant cells with intermixed spindle cell stroma. Erythrocyte lakes (secondary “aneurysmal bone cystlike” change) and xanthomatous changes within the focal collections of histiocytes may be encountered [32].

On radiographs, an expansile osteolytic lesion is typically seen. The lesion often causes collapse of the vertebral body, ranging from mild collapse to a complete vertebra plana [33]. In the sacrum, large lesions may cause destruction of the sacral foraminal lines. Extension across the sacroiliac joint is frequent [34].

**Figure 6:**
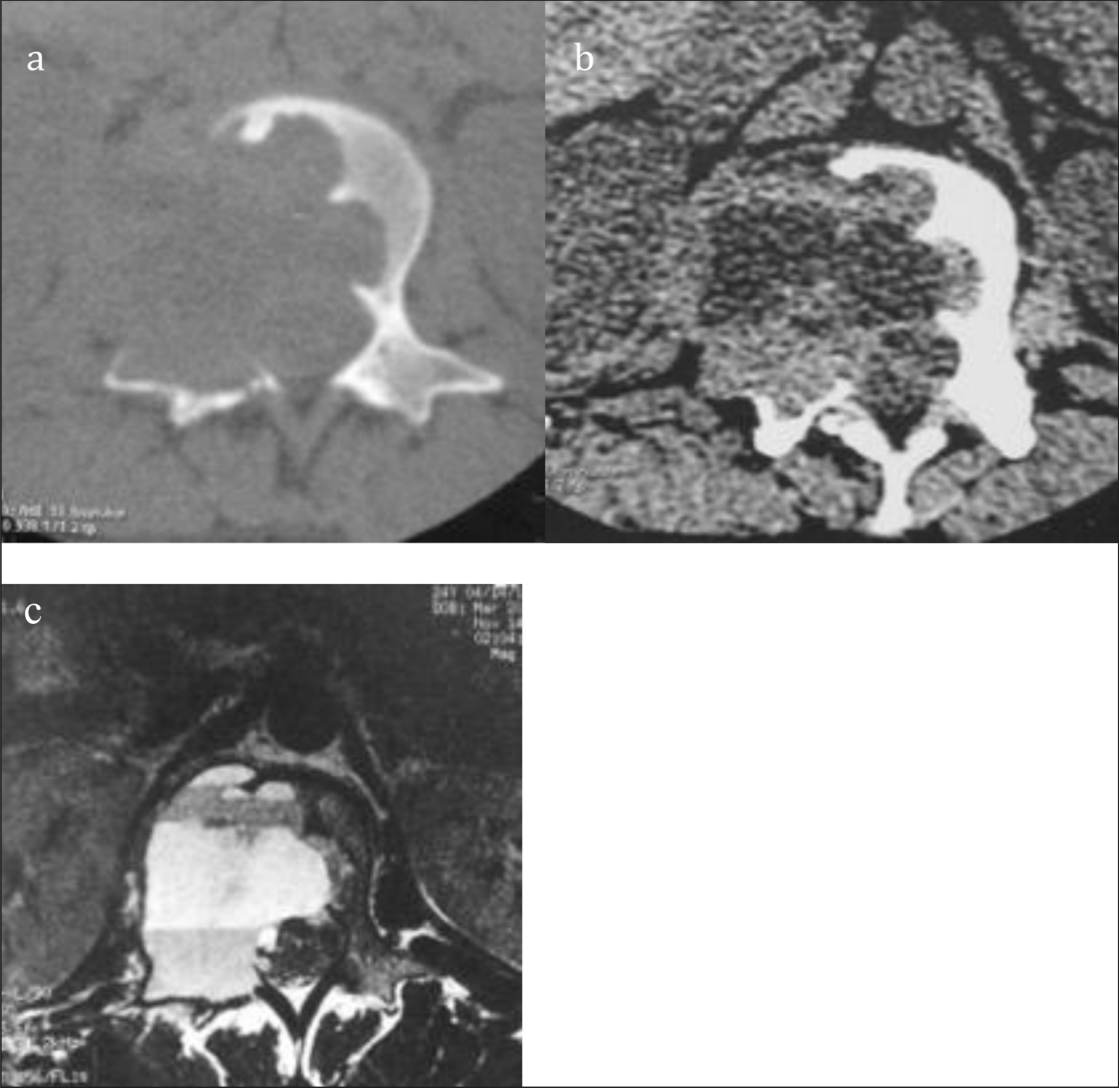
Giant Cell Tumor of L2. Axial CT scan of L2 shows a multiloculated lytic lesion **(a)** with intralesional fluid–fluid levels **(b)**. Axial T2-WI MRI confirms an expansile multiloculated lesion of the body and neural arch containing fluid-fluid levels **(c)**.

On CT, GCT has a heterogeneous density with foci of low attenuation (in keeping with haemorrhage or necrosis) with no evidence of mineralized matrix (Figure 6a, b). On MRI, GCT is of heterogeneous signal with low to intermediate signal intensity on the T1-WI and low to similar signal intensity to the normal spinal cord on the T2-WI (corresponding to fibrous components and hemosiderin). Subacute hemorrhagic foci may be of high signal intensity on the T1- and T2-WI or contain fluid–fluid levels (Figure 6c). The lesion typically shows marked enhancement after administration of intravenous gadolinium contrast [33]. The differential diagnosis of GCT includes primary aneurysmal bone cyst which usually affects the posterior elements and demonstrates a uniform cystic appearance on MRI images. GCT affects the body and there are solid components at the centre of the lesion. Another differential diagnosis is plasmacytoma. However, plastocytomas are more common in patients over 40 years of age, and cystic change is unusual.

Complete surgical resection of SGCT is the standard treatment [30]. Adjuvant medical treatment with Denosumab is promising and perhaps stand-alone therapy [35].

## Aneurysmal bone cyst

Aneurysmal bone cyst (ABC) is an expansive and hemorrhagic primary tumor, usually showing a characteristic translocation placed on 17p13 [35]. It can occur as primary bone lesion (70% of the cases), or secondary (30% of the cases) to other bone conditions (giant cell tumors, chondroblastoma, osteoblastoma, telangiectatic osteosarcoma) [36]. Primary ABC is a rare disease, accounting for 1% of primary bone tumors. Most cases are identified in the first two decades of life with a female predominance [37]. Ten to thirty percent of ABC are located in the mobile spine and comprise fifteen percent of all primary spine bone tumors [38]. ABC may occur in any segment of the spine except the coccyx. It has a predilection for the posterior elements. Vertebral bodies are occasionally affected. ABC may locally spread other vertebrae, adjacent rib, paraspinal soft tissues but intervertebral disc typically spared [39].

Clinically, ABC might even be asymptomatic but usually presents with pain and swelling. Despite being benign, they can be locally expansile and destructive, resulting in pathological vertebral fractures and neurological complications. Histologically, ABC is characterized by blood filled cavities without any endothelial lining or smooth muscle [40].

**Figure 7:**
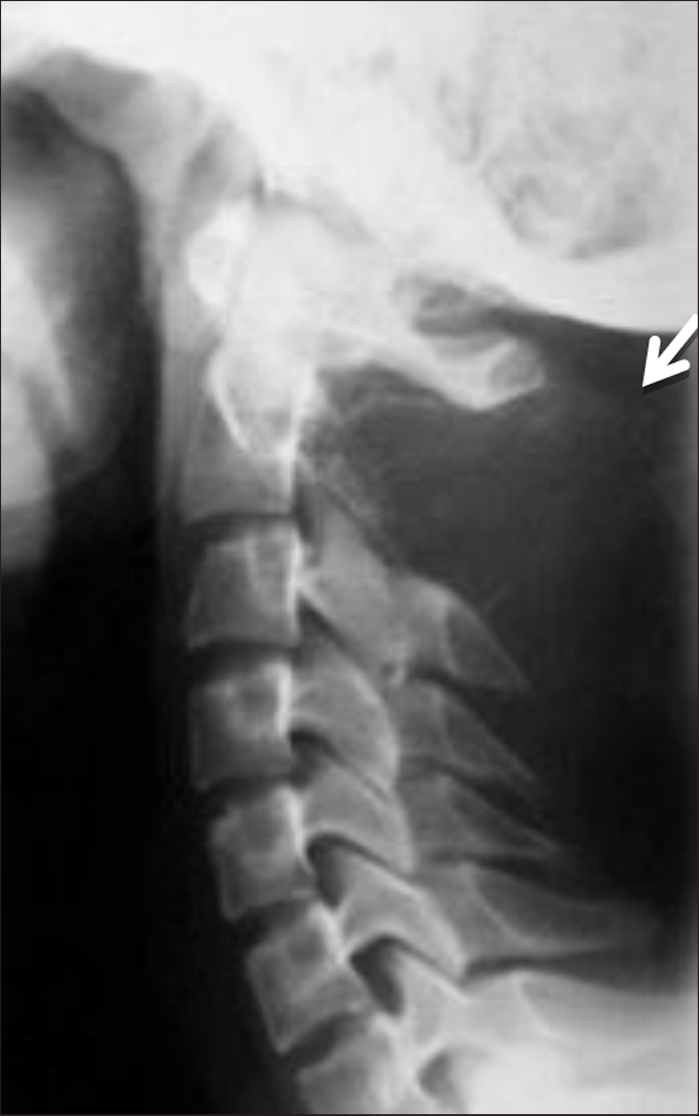
Aneurysmal bone cyst of C2. Lateral radiograph of the cervical spine shows an expansile lesion of the spinous process of C2 (arrow).

Radiographs and CT findings depend on the stage of the ABC. Eccentric bone rarefaction area is seen during the osteolytic phase (Figure 7). Active growth phase shows a subperiosteal blowout pattern. The mature stage shows the characteristic aspect of soap bubble appearance with distinct peripheral bony shell and internal bony septa and trabeculae. The healing phase is characterized by progressive calcification and ossification of the cyst[41]. CT is useful for preoperative planning. On MRI, ABC have a heterogeneous appearance on T1- and T2-WI with well defined rim of low signal intensity in the periphery. They are seen as multiseptate lesions and each lobule represents different signal characteristics. Fluid–fluid levels are typically seen in ABC.

Differential diagnosis of ABCs includes giant cell tumor, chondroblastoma, chondromyxoid fibroma, simple bone cyst, osteoblastoma, and plasmocytoma [42].

The treatment options of ABC are curettage with or without bone grafting, complete excision, arterial embolization and intralesional drug injection (steroid and calcitonin) [43].

## Fibrous dysplasia

**Figure 8:**
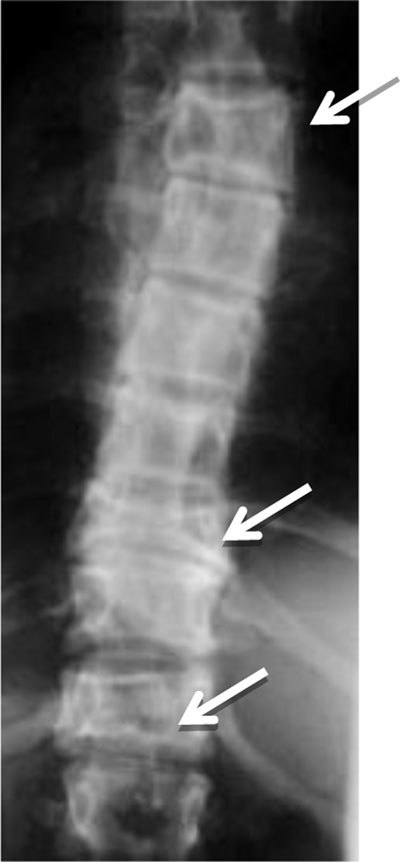
Polyostotic Fibrous Dysplasia. Radiograph shows collapse of multiple vertebral bodies (T6, T10, T12) with a ground glass matrix.

Fibrous dysplasia (FD) is a benign medullary fibro-osseous lesion, which may be monostotic or polyostotic. Monostotic FD is more common than polyostotic FD. However, polyostotic disease occurs in the spine more frequently [44]. FD affects primarily children and adolescents and is estimated to involve the spine in only 2.5% of all cases. FD may affect each segment of the spine, with the highest prevalence in the lumbar region. Contiguous invasion of the posterior aspect of the ribs may be seen [45]. The most common symptoms of polyostotic FD are pain and scoliosis [46]. Spinal compression syndromes may result from pathologic fractures. Histopathological findings classically include retracted osteoblasts and woven abnormal fibrous bony matrix [47]. Radiographic findings include “ground-glass appearance,” areas of marginal sclerosis adjacent to lytic regions with a narrow transitional zone and pathological fractures (Figure 8) [48]. CT shows a mildly expansile lesion with a “blown-out” cortical shell or a lytic lesion with a sclerotic rim [49]. MRI typically shows intermediate to low signal intensity on T1-WI and intermediate to high on T2-WI. Benign lesions are well-demarcated without cortical bone disruption or soft-tissue extension. Malignant transformation is very rare and should be suspected in case of cortical destruction with associated soft-tissue mass [50]. Isolated vertebral involvement in polyostotic form may simulate metastasis and multiple myeloma [51].

The treatment of monostotic fibrous dysplasia of the spine varies widely, from wait-and-see policy to biopsy and surgical resection.

## Langerhans’ cell histiocytosis

Langerhans’ cell histiocytosis (LCH) is a rare disorder characterized by proliferation of histiocytes or macrophages [52]. LCH can affect patients of any age, although it most commonly present in patients younger than 15 years, with a male predominance. Spinal involvement is seen in 10–15% with predilection in the thoracic spine, followed by the lumbar and cervical spine. Vertebral bodies are involved much more commonly than the posterior elements [53].

The most common presenting symptom is dull pain. Neurologic complications are rare and usually mild [54].

Histopathologically, eosinophilic granulomas display a characteristic “tennis racquet”-shaped “Birbeck granule” in the cytoplasm [49].

**Figure 9:**
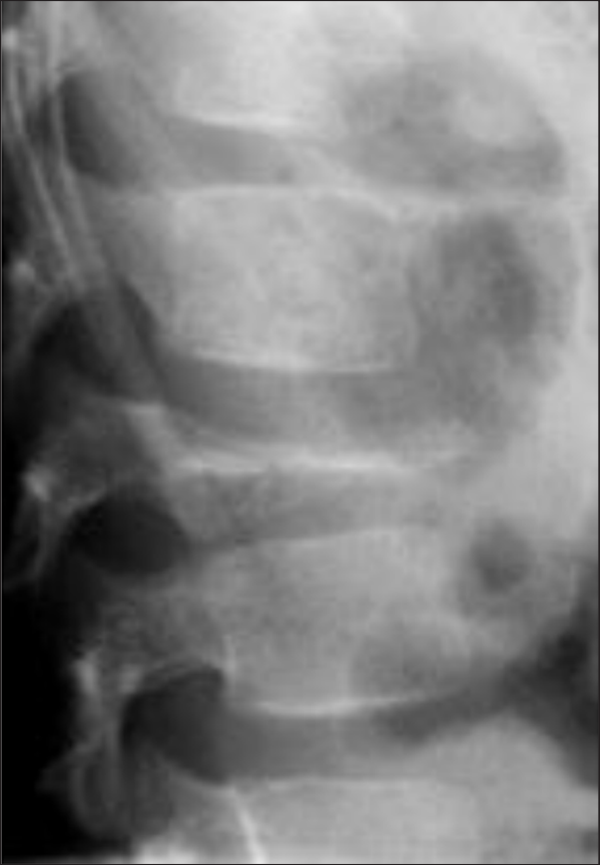
Eosinophilic granuloma (solitary form of Langerhans’ cell histiocytosis). Lateral radiograph shows collapse of the vertebral body L1 (vertebra plana).

Radiographs show an anterior wedging with a characteristic “vertebra plana” appearance (Figure 9). Epidural soft-tissue extension may be seen on CT or MRI. MRI shows preservation of the adjacent disk spaces which helps differentiating the lesion from infection.

The differential diagnosis include other destructive lesion including Ewing sarcoma, lymphoma, leukemia, and metastasis. Trauma should be considered as well in case of vertebra plana [53].

Therapeutic options range from clinical/radiological monitoring to cervical or lumbar orthoses, CT-guided intralesional infiltration of corticosteroids, surgical curettage and bone grafting, or even radical resection of the affected bone [55].

## Dense Bone Island (Enostosis)

**Figure 10:**
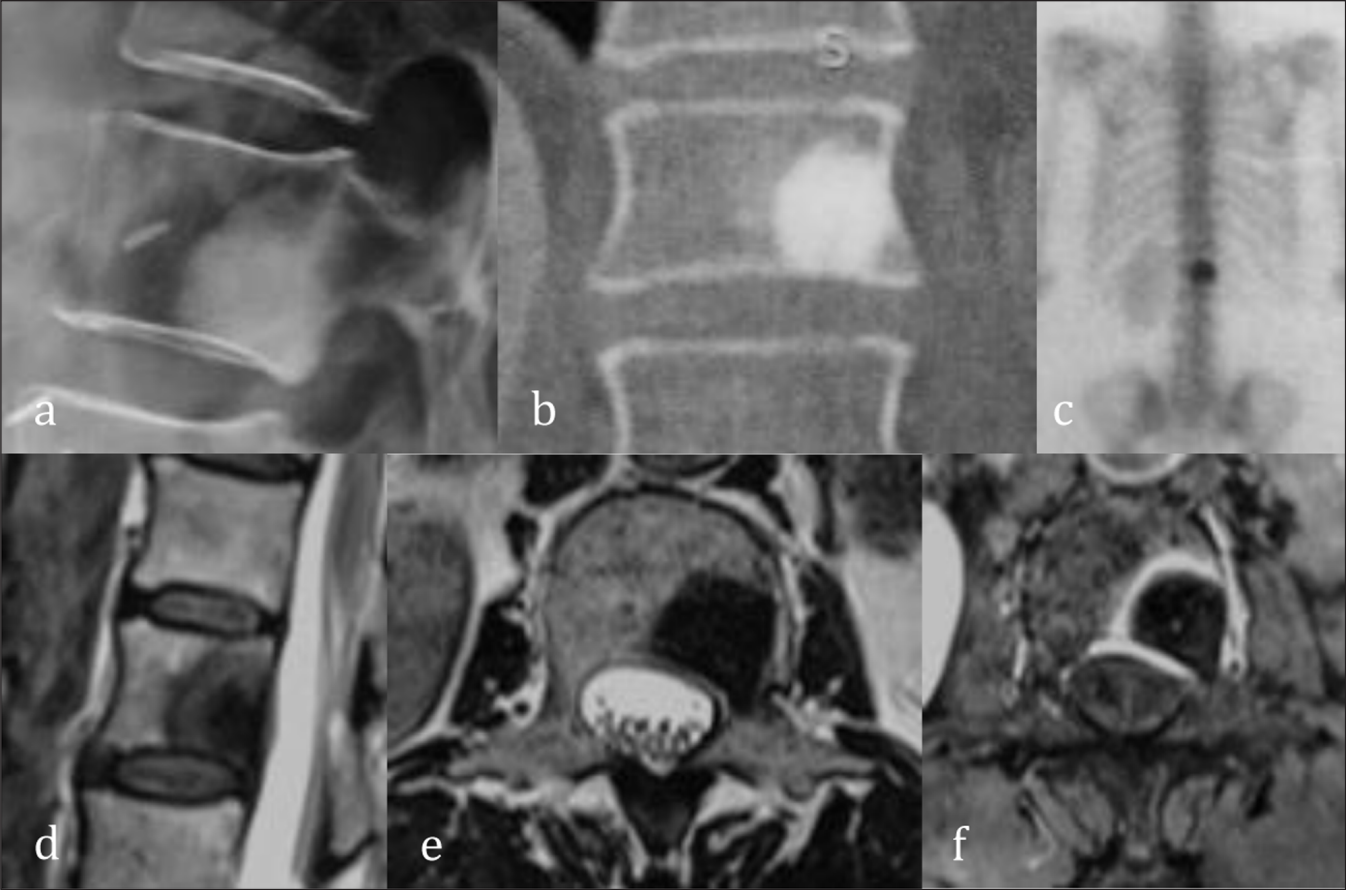
Enostosis of L1. Radiograph **(a)** and coronal reformatted CT **(b)** show a focal area of dense bone similar to cortical bone. Bone scintigraphy **(c)** show an avid uptake. Sagittal T2-WI **(d)**, axial T2-WI **(e)** and axial T1-WI after administration of gadolinium contrast **(f)** show central low signal intensity surrounded by intermediate T2 signal and peripheral enhancement.

Enostoses are relatively common lesions with an incidence of up to 14% in cadaveric studies. These lesions are found in all age groups with no significant gender predilection [56]. Histologically, enostosis represent a developmental hamartomatous cortical bone embedded within the trabecular network of the medullary cavity [29]. They are related to dysplasias of endochondral bone formation [56]. Radiography and CT demonstrate a homogeneously dense, sclerotic focus of cancellous bone with distinctive radiating spiculations at their periphery, resulting in a so-called “brush border”. Enostoses are of low signal intensity on T1 and T2-WI. They may show increased activity on bone scintigraphy in less than 10% of cases (Figure 10) [54]. The main differential diagnosis is osteoblastic metastasis. CT attenuation measurements may be helpful to distinguish untreated osteoblastic metastases from enostoses [57]. Biopsy should be considered if the lesion increases in diameter by more than 25% during a six-month period [58].

## Conclusion

The combination of age, location, and imaging appearance is often needed for a correct diagnosis of benign spinal tumors. Careful analysis of semiological signs may allow precise characterization of most benign bone tumors of the spine and helps in differentation from malignant primary spinal tumors, metastases or other non tumoral lesions.
